# Impact of *HOMER2* frameshift extension variant on auditory function and development

**DOI:** 10.1007/s00109-025-02556-7

**Published:** 2025-06-14

**Authors:** Eunjung Han, Ju Ang Kim, Saemi Park, Jin Hee Han, Min Young Kim, Yehree Kim, Ngoc-Trinh Tran, Bong Jik Kim, June Choi, Byung Yoon Choi

**Affiliations:** 1 College of Medicine, Korea University Ansan Hospital, Korea University, Ansan, Republic of Korea; 2 Zebrafish Translational Medical Research Center, Korea University, Seoul, Republic of Korea; 3 Department of Otorhinolaryngology, College of Medicine, Seoul National University Hospital, Seoul National University, Seongnam, Republic of Korea; 4 Chungnam National University Sejong Hospital, Sejong, Republic of Korea

**Keywords:** HOMER2, Sensorineural hearing loss, Frameshift extension variant, Zebrafish, Cardiac anomaly

## Abstract

The *HOMER2* gene, crucial for synaptic signaling and calcium homeostasis in the auditory system, is linked to sensorineural hearing loss (SNHL), with its variants contributing to severe SNHL in older adults, often necessitating cochlear implants in their 60 s or 70 s. In this study, we identified a novel frameshift extension variant, c.1033delC (p.Arg345Glufs*64; p.R345Efs*64), which introduces a significantly longer protein extension than previously reported extension variants, in a patient in their sixties presenting with progressive profound SNHL. To investigate the pathogenic potential of this variant, we employed molecular modeling and zebrafish models, comparing wild-type HOMER2, a hypothetical p.R345* variant involving alteration of the most C-terminal 10 amino acids, and the patient-derived p.R345Efs*64 variant. AlphaFold2 predicts that the p.R345Efs*64 variant causes significant structural changes in the HOMER2 EVH1 domain, disrupting interactions with Cdc42 and contributing to SNHL. Zebrafish models show that this variant, which combines truncation and extension features, impairs neuromast hair cell function and exacerbates auditory phenotypes, while also increasing cardiac anomalies. In comparison, the p.R345* variant showed an obvious milder impact. Our findings suggest that the pathogenic effect of the p.R345Efs*64 variant is more driven by the extension beyond the stop codon. Here we report that a novel frameshift extension variant of *HOMER2*, which arises as a causative gene in elderly patients with profound SNHL, highlighting the need for genetic diagnosis in this population. Our findings reveal a solid pathogenic gain-of-function effect related to the long extension to the C-terminal of *HOMER2*, and a possible link to cardiac anomalies.

**Supplementary Information:**

The online version contains supplementary material available at 10.1007/s00109-025-02556-7.

## Key messages

- We report a novel and unique frameshift extension variant, c.1033delC, in *HOMER2*.
- This *HOMER2* variant leads to profound deafness in people in their 60s or later.
- The extended 63 amino acids of the variant may disrupt HOMER2’s EVH1 domain.
- The variant impairs morphological development and auditory function in zebrafish.

**Supplementary Information:**

The online version contains supplementary material available at 10.1007/s00109-025-02556-7.

## Introduction

Sensorineural hearing loss (SNHL) is a prevalent sensory disorder affecting millions of individuals worldwide, with both genetic and environmental factors contributing to its etiology. Among the numerous genes implicated in auditory function, the *HOMER2* gene has emerged as a significant player due to its role in synaptic signaling and intracellular calcium homeostasis within the auditory system [1, 2]. Specifically, *HOMER2* encodes a scaffolding protein that interacts with various receptors and channels, facilitating the proper transmission of auditory signals and maintaining cochlear function. Thus, variants in *HOMER2* have been associated with disrupted synaptic connectivity and impaired auditory processing, leading to SNHL [1, 3]. This gene also has a unique feature as it could become a key factor in the progression of severe SNHL in old age, potentially requiring cochlear implantation (CI) when patients reach their 60 s or 70 s.

Previous studies have identified only a handful of variants within the *HOMER2* gene that result in diverse phenotypic manifestations, ranging from mild to profound SNHL, depending on age at ascertainment [3, 4]. These variants often lead to truncated or dysfunctional proteins that fail to support normal synaptic architecture and signaling pathways [4]. Notably, nonsense and frameshift variants that particularly evade nonsense-mediated decay have been extensively studied, revealing critical insights into the dominant negative molecular mechanisms underpinning *HOMER2*-related auditory deficits [5]. However, the entire variant spectrum of *HOMER2* variants and their corresponding pathogenic potentials remain incompletely understood, necessitating further investigation to elucidate their impact on auditory function and overall organismal health.

In this context, we have identified a novel frameshift extension variant, c.1033 delC (p.R345Efs*64), in a patient in her sixties presenting with progressive profound SNHL at our institution. This long extension variant can lead to protein instability, misfolding, and aberrant interactions, potentially culminating in severe functional impairments. The unprecedented length of the extension in p.R345Efs*64 that far exceeds the recently reported 10 amino acid extension found in simpler extension variants [4], raises critical questions regarding its pathogenicity and the resultant phenotypic consequences, both in auditory function and broader physiological contexts. Unlike a simple non-stop extension variant, our variant has an additional complexity introduced by the frameshift involving 10 amino acids at the original C-terminus. Therefore, we aimed to characterize the pathogenic potential of this more extensive and structurally altered variant. To assess the pathogenic potential of the p.R345Efs*64 variant, we employed a molecular modeling and a zebrafish (*Danio rerio*) model system, where wild-type (WT) HOMER2, p.R345*-*HOMER2* generated artificially to mimic loss-of-function effects, and the patient-derived p.R345Efs*64-HOMER2 was tested and compared.

## Materials and methods

### Subject and phenotyping of clinical features

A 61-year-old woman (SB1190-1923) was ascertained to have profound SNHL mandating CI by pure tone audiogram. History-taking revealed the natural course of SNHL of SB1190-1923. During the CI operation, electrically evoked compound action potential (ECAP) thresholds were recorded across all channels using the automated neural response telemetry measurement mode, following the methodology described in prior studies [6, 7]. Preoperative and postoperative evaluations of auditory rehabilitation were performed using the speech recognition test (one syllable, two syllables, and sentence), as previously described [8, 9]

### Genotyping of the subject

Genomic DNA was extracted from blood samples obtained from SB1190-1923 for genetic analysis. An initial screen was performed for 11 recurrent variants across five deafness-related genes, as previously reported [10, 11], but no candidate variants were identified to account for the hearing loss observed in SB1190. Following this, exome sequencing (ES) was conducted, and bioinformatics filtering techniques were applied to narrow down potential candidate variants (Supplementary Fig. 1) [12–15]. Thirty-four genes were eliminated from further analysis after exome sequencing due to their classification as benign or variants of uncertain significance (VUS), indicating a low likelihood of clinical relevance (Supplementary Table 1). The causative variant identified through this approach was subsequently validated via Sanger sequencing.

### Molecular modeling

The protein sequence of HOMER2 [NP_955362], along with the variants p.R345Efs*64 and p.R345*, are presented in the Supplementary Information (Supplementary Table 2). We utilized AlphaFold2 (V2.1) [16] to predict the structures of wild-type HOMER2 and its variant forms (p.R345Efs*64 and p.R345*). To align the HOMER2 wild-type and variant structures, we employed TM-align [17]. Additionally, MM-align [18] was used to align HOMER2 with the dimer form of Cdc42. For visualization and analysis of the aligned 3D protein structures, Mol*3D Viewer (Mol* Plugin 4.4.1) was used.

### Zebrafish housing and experimental procedures

Adult zebrafish were maintained in a controlled aquatic facility under a 14-h light and 10-h dark cycle, enabling proper identification for subsequent breeding studies. At 2 months post-fertilization, the fish were sexed and separated for mating. Embryos were incubated at 28.5 °C, and larvae were utilized for experiments at 3- and 6-day post-fertilization (dpf).

### Cloning of human HOMER2 variants and mRNA synthesis

To generate human *HOMER2* mRNA, wild-type (WT) and two variants (p.R345* and R345Efs*64) were cloned into the PCS22 + vector (Novo Pro, V011726). The *HOMER2* tagged ORF clone (NM_199330) was obtained from OriGene (RC223154). Cloning was performed by inserting the WT and variant cDNAs into the PCS22 + vector following restriction enzyme digestion and ligation. Correct insertions were confirmed by Sanger sequencing.

For mRNA synthesis, the linearized recombinant plasmids were transcribed using the mMESSAGE mMACHINE™ SP6 Transcription Kit (Invitrogen, AM1340), following the manufacturer’s protocol. The reaction was incubated at 37 °C, and mRNA was purified via lithium chloride precipitation. The quality and quantity of the synthesized mRNA were evaluated by spectrophotometry and agarose gel electrophoresis. Purified mRNA was stored at – 80 °C until use.

### mRNA microinjection into zebrafish embryos

Microinjections were performed on zebrafish embryos at the 1-cell stage to assess the function of the *HOMER2* mRNA variants. The synthesized *HOMER2* mRNA (WT, p.R345*, and p.R345Efs*64) was diluted to a final concentration of 200 ng/µL. To confirm successful injections, red fluorescent protein (RFP) mRNA was co-injected at the same concentration of 200 ng/µL. The mRNA mixtures were prepared before injection. The mRNA mixture was injected directly into the yolk of 1-cell stage zebrafish embryos using a calibrated glass capillary needle under a stereomicroscope. After injection, embryos were incubated at 28.5 °C in E3 medium and screened for successful injection by observing RFP fluorescence. Embryos showing uniform RFP expression were selected for further analysis.

### Staining and imaging of lateral line hair cells

Lateral line hair cells in zebrafish larvae were labeled using YO-PRO-1 iodide (Life Technologies, Y3603), a fluorescent marker for cell viability. Larvae were incubated in a 1:1000 dilution of YO-PRO-1 in phosphate-buffered saline for 1 h at room temperature, allowing for selective staining of the hair cells. Following incubation, larvae were thoroughly washed with embryo medium to remove excess dye and subsequently anesthetized using tricaine (MS-222; Sigma-Aldrich, A5040) to ensure immobilization during imaging. Four neuromasts were selected as the primary focus for cell counting, consistent with previous protocols. Fluorescence imaging was conducted using a Nikon ECLIPSE Ni-U microscope (Nikon, Japan), capturing high-resolution images of stained neuromast cells.

### FM1-43 uptake

Neuromast hair cell activity was assessed by exposing zebrafish larvae to the fluorescent dye FM™ 1–43 Dye (Invitrogen, T3163), a known marker for active mechanosensory hair cells. Larvae were incubated in a 3 µM solution of FM1-43 for 30 s at room temperature, followed by three washes in embryo medium to remove excess dye. After washing, larvae were anesthetized with tricaine (Sigma-Aldrich) to ensure immobility during imaging. Imaging was performed using a Nikon C5U-X1 confocal microscope (Nikon, Japan) to capture Z-stack images of stained neuromasts. Fluorescence intensity in the neuromast cells was analyzed with specific regions of interest defined around individual neuromasts.

### Startle reflex test

The startle reflex test was conducted following the locomotor behavior analysis, using a method previously described in our earlier study [19]. After recording larvae behavior in a static state for 5 min, followed by a 10-min rest interval, larvae were subjected to mechanical stimulation by tapping, implemented within the DanioVision system (Noldus Information Technology, The Netherlands). We measured latency, distance moved. A valid startle response was defined as an initiation of movement within 0.5 s of tapping, with the larvae moving more than 0.6 mm during the reflex. Behavioral data were analyzed using EthoVision XT software (version 17.5).

### Data analysis and statistics

All data were analyzed using Prism 10 software. Quantitative results are presented as mean ± standard error of the mean (SEM). For comparisons between multiple groups, one-way analysis of variance (ANOVA) was performed, followed by post-hoc Tukey’s multiple comparisons test. Chi-squared tests were used for categorical data comparisons, such as the incidence of morphological abnormalities.

## Results

### Phenotype and genotype of SB1190

**Fig. 1:**
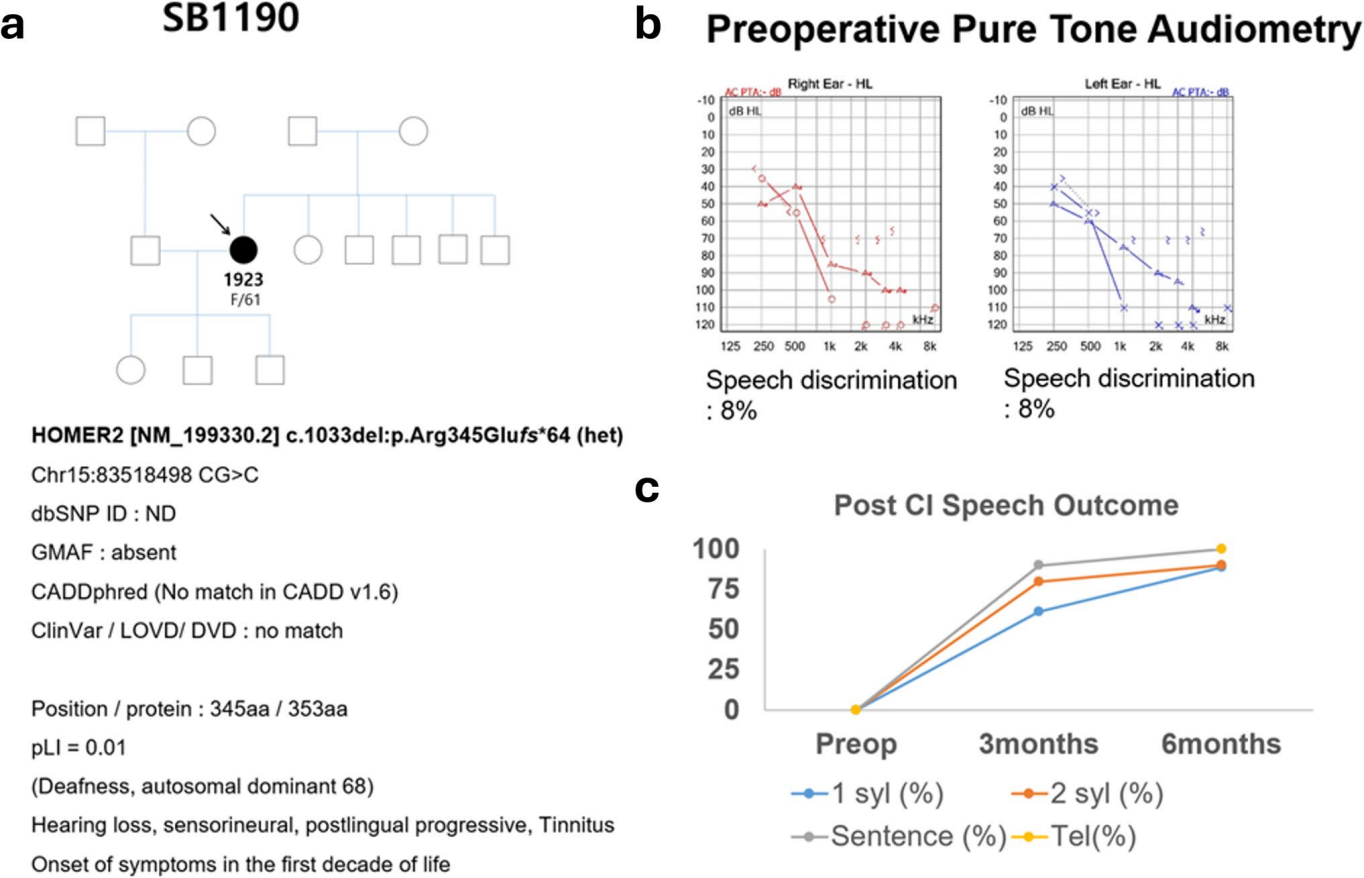
Pedigree, audiometric profile, genetic analysis, and post-cochlear implantation speech outcomes from SB1190-1923 (F/61). **a** Pedigree of SB1190: the proband (F/61) carries the previously unreported, novel *HOMER2* variant (c.1033 del (p.Arg345Glufs*64)) which is associated with DFNA68. Low pLI score (0.01) of *HOMER2* suggests that alteration of *HOMER2* exerts a pathogenic effect via a mechanism other than loss-of-function. Both parents of SB1190-1923 passed away in their early 70 s without showing any signs of significant hearing loss, and none of the SB1190-1923’s children exhibited any symptoms of hearing loss. **b** Preoperative pure tone audiograms show profound down-sloping sensorineural hearing loss with 8% speech discrimination score bilaterally. **c** Post-Cochlear Implantation speech outcome speech recognition improves significantly at 3- and 6-months post-implantation across various syllable and sentence conditions. *F*, female; *pLI*, intolerant; *CI*, cochlear implantation

SB1190-1923 began to experience more severe progressive SNHL in the high-frequency range starting in their 50 s. Ten years after the onset of significant SNHL, SB1190-1923 had developed profound deafness with a speech discrimination score of only 8% **(**Fig. 1**)**. ES identified a potentially causative, novel variant of *HOMER2* (c.1033 del:p.R345Efs*64) in a single heterozygous state, and the variant status was confirmed with Sanger sequencing.

The patient’s SNHL phenotype was matched perfectly with previous reports in the literature related to *HOMER2* [1, 3, 5, 20, 21] (Table 1 and Fig. 1). This variant, which we categorize as a “frameshift extension variant,” features a cytosine deletion at Chr15:83,518,499, leading to a frameshift that alters R345 to E(Glu) and results in the addition of 63 amino acids after R344.

**Table 1:** *HOMER2* variants reported thus far in the literature and this study

Subject ID (or dbSNP ID)	Genomic position: change (GRCh37/hg19)	HGVS		Zygosity/inheritance	In silico predictions		Alternative allele frequency		ACMG/AMP 2018 guideline		Reference (PMID)
		Nucleotide change	Amino acid change		CADD Phred	REVEL	KOVA	GMAF (gnomAD)	Criteria	Classification	
SB1190-1923 (dbSNP ID: absent)	Chr15:83,518,498 CG > C	[NM_199330] c.1033 del	[NP_955362] p.Arg345Glu*fs*Ter64	Heterozygote	NA	NA	Absent	Absent	PS3_Moderate, PM2, PM4	Likely Pathogenic	This study
(rs1403441552)	Chr15:83,518,499 G > A	[NM_199330] c.1033 C > T	[NP_955362] p.Arg345 Ter		36.00	NA	Absent	A = 0.000008 (2/248840, GnomAD_exome)	PM2	Uncertain Significance	This study (Artificial variant)
(rs864309524)	Chr15:83,523,493 C > G	[NM_004839] c.554G > C [NM_199330] c.587G > C	[NP_004830] p.Arg185Pro [NP_955362] p.Arg196Pro	Heterozygote/ AD	26.00	0.713	Absent	Absent	PS1, PS3_Moderate, PM2, PP1_Strong	Pathogenic	[1]
Family-202 (rs2051382723)	Chr15:83,519,938 T > TG	[NM_199330] c.840 dup	[NP_955362] p.Met281His*fs*Ter9	Heterozygote/ AD	NA	NA	Absent	Absent	PS3_Moderate, PM2, PP1_Strong	Likely Pathogenic	[4]
Family 32 (dbSNP ID: absent)	Chr15:83,523,482 CTGTGGT > C	[NM_199330] c.592_597 del	[NP_955362] p.Thr198_Thr199 del	Heterozygote/ AD	NA	NA	Absent	Absent	PM2	Uncertain Significance	[19]
Family S1074 (rs2051382999)	Chr15:83,519,942 CTGAGG > C	[NM_199330] c.832_836 delCCTCA	[NP_955362] p.Pro278 Ala*fs*Ter10	Heterozygote/ AD	NA	NA	Absent	Absent	PM2, PP1	Uncertain Significance	[3]
teenage boy (rs771053430)	Chr15:83,544,131 G > A	[NM_199330.2] c.188 C > T	[NP_955362.1] p.Pro63Leu	Heterozygote/ AD	29.10	0.766	Absent	A = 0.000004 (1/249116, GnomAD_exome)	PM2, PP3	Uncertain Significance	[20]
SU340	Chr15:83,518,468 T > C	[NM_199330] c.1064 A > G	[NP_955362] p.Ter355 Trp*ext*Ter10	Heterozygote/ AD	17.79	NA	Absent	Absent	PS3_Moderate, PM2, PP1_Strong	Likely Pathogenic	[5]

The human *HOMER2* gene has two types of transcripts, each encoding a different protein: one based on the long transcript (NM_199330, encoding NP_955362 with a length of 354 amino acids) and the other on a shorter transcript (NM_004839, encoding NP_004830 with a length of 343 amino acids). Among the variants listed in the table, for the variant discussed in the [1] paper, the nomenclature was checked and added based on the long transcript due to the difference in transcript type. *AD*, autosomal dominant; *GMAF*, global minor allele frequency; *NA*, not available; *ACMG*, the American College of Medical Genetics and Genomics; *AMP*, the Association for Molecular Pathology. Web resources: HGVS: Human Genome Variation Society (https://www.hgvs.org/), CADD: Combined Annotation Dependent Depletion (https://cadd.gs.washington.edu/), REVEL: Rare Exome Variant Ensemble Learner (https://sites.google.com/site/revelgenomics/), KOVA: Korean Variant Archive for a reference database of genetic variations in the Korean population (https://www.kobic.re.kr/kova/), gnomAD: The Genome Aggregation Database (https://gnomad.broadinstitute.org/)

Based on the previously reported sixth variant [4], specifically the nonstop extension variant identified as pathogenic, we were reasonably confident that our variant, which results in a longer extension, would also be pathogenic. Therefore, we aimed to determine whether the pathogenic effect of our variant was primarily driven by the alteration of the 10 amino acids at the C-terminus or by the extension of 53 additional amino acids beyond the stop codon. To investigate this, we conducted molecular modeling and functional studies in zebrafish, comparing a hypothetical variant where the C-terminal 10 amino acids were truncated (*HOMER2* p.R345*) with our actual frameshift extension variant (*HOMER2* p.R345Efs*64).

### Protein modeling prediction

**Fig. 2:**
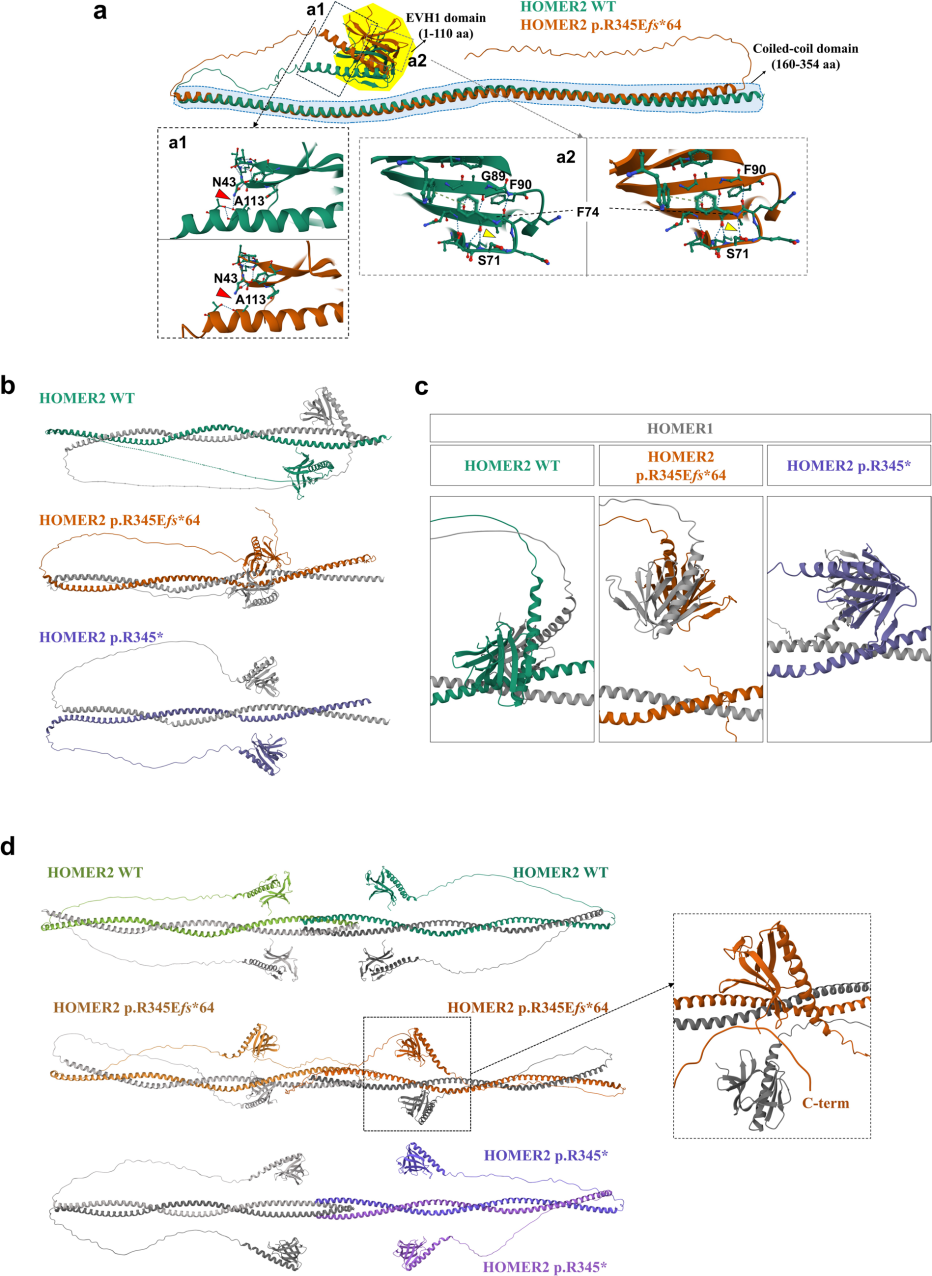
Predicted 3D structures of HOMER2 WT and HOMER2 p.R345Efs*64 using AlphaFold2. **a** Structural alignment of HOMER2 WT and HOMER2 p.R345Efs*64. The EVH1 domain is highlighted in the yellow area, and the coiled-coil (CC) domain is marked by the blue dashed line. **a1** The hydrogen bond between N43 and A113 is disrupted in HOMER2 p.R345Efs*64 compared to HOMER2 WT. **a2** The β-sheet structure in HOMER2 p.R345Efs*64 extends to F90, and a hydrogen bond forms between F74 and S71, which is absent in HOMER2 WT. **b** The structure of the HOMER1 [NP_004263.1] and HOMER2 dimer is shown. HOMER1 is depicted in gray, while HOMER2 WT, HOMER2 p.R345Efs*64, and HOMER2 p.R345* are represented in green, brown, and purple, respectively. The coiled-coil region of HOMER2, which can interact with other proteins, is located at amino acids 307–329 in the WT and is shifted to amino acids 275–297 in the HOMER2 variants. **c** Structural changes in the EVH1 domain were detected in the HOMER1 WT and HOMER2 variants dimer. The distance between each EVH1 domain of HOMER1 and HOMER2 p.R345Efs*64 was reduced (**b**) compared to the WT, which is shifted vertically. In HOMER1 WT and HOMER2 p.R345* dimer, the distance between each EVH1 was increased (**b**) and slightly shifted vertically. **d** The predicted tetramer structures were obtained using HOMER1 and HOMER2. Two HOMER1 proteins were combined with two HOMER2 WT, two HOMER2 p.R345Efs*64, or two HOMER2 p.R345* molecules. In the HOMER1 and HOMER2 p.R345Efs*64 tetramer, the location of the EVH1 domain of HOMER1 was altered, and the C-terminal of HOMER2 approached the EVH1 domain of HOMER2 (dashed black box). In the HOMER1 and HOMER2 p.R345* tetramer, HOMER1 and HOMER2 formed homodimers, and each homodimer formed a tetramer, unlike the other configurations. *WT*, wild-type; *R*, arginine; *E*, glutamate; *fs*, frameshift; *EVH1*, Ena/Vasp homology domain 1; *F*, phenylalanine; *S*, serine; *N*, asparagine; *A*, alanine

The HOMER2 variant p.R345Efs*64 causes a frameshift extension affecting the C-terminal coiled-coil (CC) domain. The CC domain in the C-terminal region of HOMER2, near which p.R345 is located, is known as essential for interactions between Homer proteins [22]. In contrast, the EVH1 (Ena/Vasp Homology domain 1) domain, located relatively closer to the N-terminus of HOMER2, plays a crucial role in mediating interactions with other proteins, including the IP3 receptor and C/EBPβ [23, 24]. Structural predictions using AlphaFold2 suggest that the p.R345Efs*64 variant causes structural changes in the EVH1 domain (Fig. 2a), rather than directly affecting the CC domain. When predicting dimer structures with HOMER1 and HOMER2, the position of the EVH1 domain in the two variants (p.R345* and p.R345Efs*64) HOMER2 proteins was found to be significantly different compared to the WT protein (Fig. 2b). Upon observing the dimer structures from the side, it was noted that the EVH1 domains were more vertically shifted in the dimer with HOMER2 p.R345Efs*64 than with WT and p.R345* variant (Fig. 2c).

As previously reported, HOMER proteins form tetramers [25]. Similarly, tetramer structures were predicted using HOMER1. Interestingly, HOMER2 WT, HOMER2 p.R345* and HOMER2 p.R345Efs*64 formed dimers with HOMER1, and two of these dimers formed a tetramer unlike with HOMER2 p.R345* (Fig. 2d). Notably, a significantly interfered EVH1 domain was detected in the HOMER2 p.R345Efs*64-containing tetramer (Fig. 2d, black dashed box).

### Zebrafish study results

**Fig. 3:**
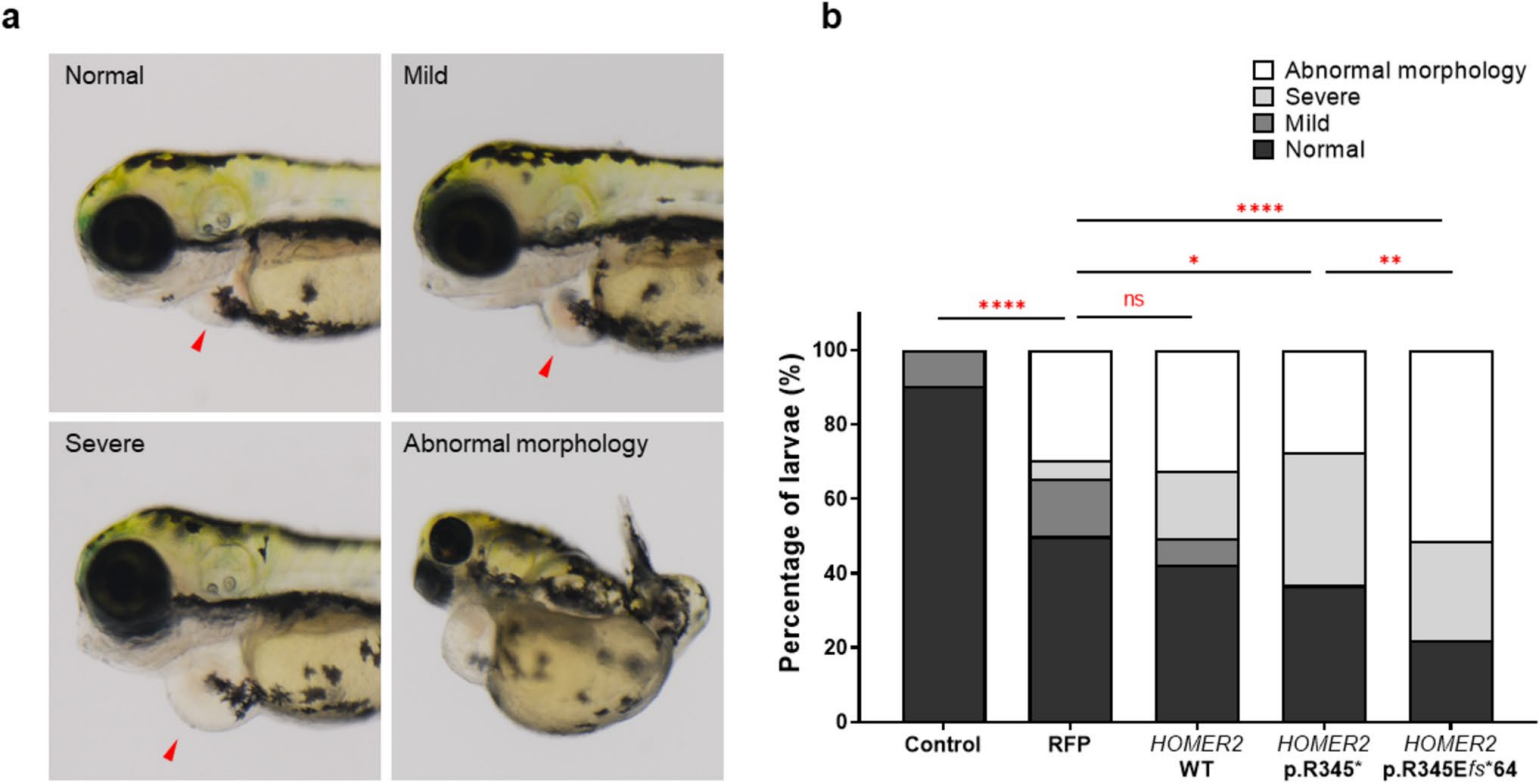
Cardiac and morphological defects in zebrafish larvae injected with *HOMER2* mRNA. **a** Representative images of zebrafish larvae at 3 dpf displaying varying degrees of heart malformations. The larvae were categorized into four groups: normal, mild, and severe heart defects based on the degree of cardiac enlargement, and an additional group with general abnormal morphology. The categories include normal, mild, and severe heart defects, with red arrowheads marking the regions of cardiac deformity. Additionally, larvae with abnormal overall morphology are shown. **b** Bar graph illustrating the distribution of larvae with heart and morphological abnormalities in the different experimental groups: uninjected control, RFP control, *HOMER2* WT, *HOMER2* p.R345*, and *HOMER2* p.R345Efs*64. Chi-squared analysis was used to compare the proportions of defects between groups, with significant differences indicated. (****p < 0.0001, ***p < 0.001, **p < 0.01, *p < 0.05). *n* = 100 per group. *dpf*, days post-fertilization; *RFP*, red fluorescent protein; *WT*, wild-type; *R*, arginine; *E*, glutamate; *fs*, frameshift

Through protein prediction modeling, it was predicted that both p.R345Efs*64 and p.R345* have pathogenic effects. Therefore, we decided to test both variants in a zebrafish model. Following the injection of *HOMER2* mRNA of WT and both variants (hypothetical and patient-derived) into zebrafish embryos, larvae were observed to have heart malformations and overall morphological defects at 3 dpf. (Fig. 3a). The distribution of four categories was analyzed across five groups including *HOMER2* variants. The percentage of larvae exhibiting heart malformations and abnormal morphology increased progressively in the *HOMER2* variants group. Specifically, the proportion of malformations in both *HOMER2* variant groups was significantly increased compared to the RFP control group (1.63-fold in p.R345* and 1.78-fold in p.R345Efs*64) (Fig. 3b), indicating a stronger effect of these variants on cardiac development.

**Fig. 4:**
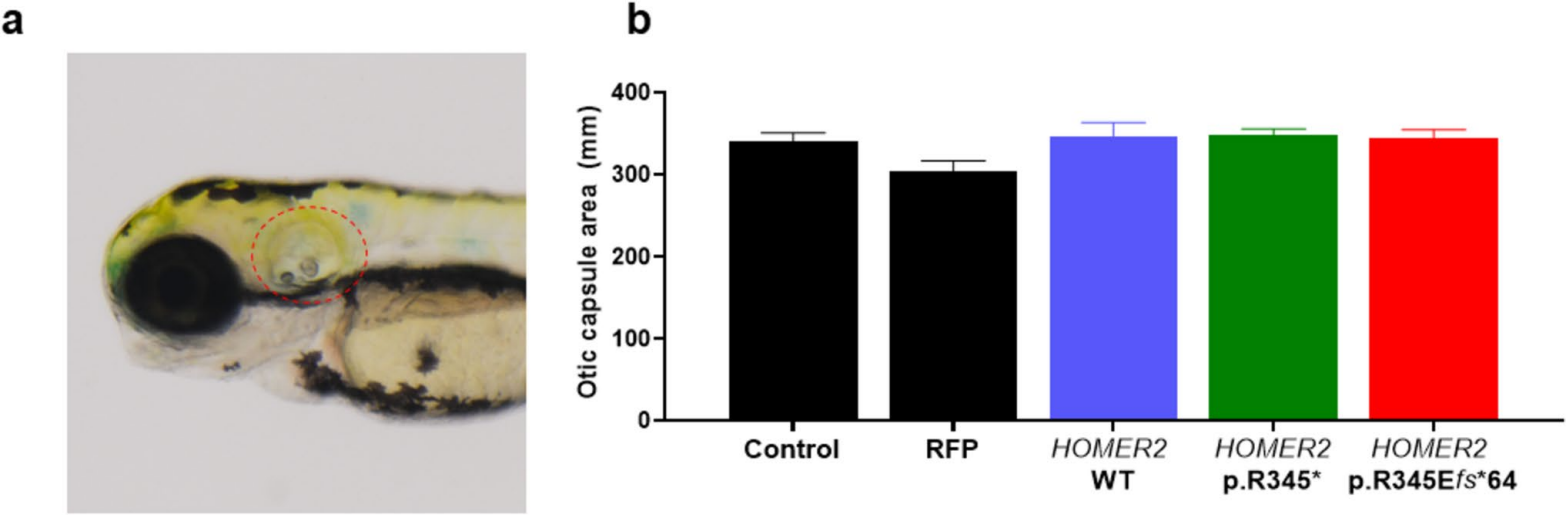
Otic capsule area in zebrafish larvae at 3 days post-fertilization.** a** Representative image of 3 dpf zebrafish larvae, with the otic capsule indicated by a red dashed circle. **b** Bar graph showing the measured otic capsule area in control, RFP control, *HOMER2* WT, *HOMER2* p.R345*, and *HOMER2* p.R345Efs*64 groups. No statistically significant differences were observed between groups (*p* > 0.05). *n* = 15 per group. *dpf*, days post-fertilization; *RFP*, red fluorescent protein; *WT*, wild-type; *R*, arginine; *E*, glutamate; *fs*, frameshift

To assess the potential effects of HOMER2 mRNA variants on auditory system development, the otic capsule area was measured (Fig. 4a). Quantification of the otic capsule area across five groups and its statistical analysis indicated no significant impact of HOMER2 variants on the size of the otic structure (Fig. 4b).

**Fig. 5:**
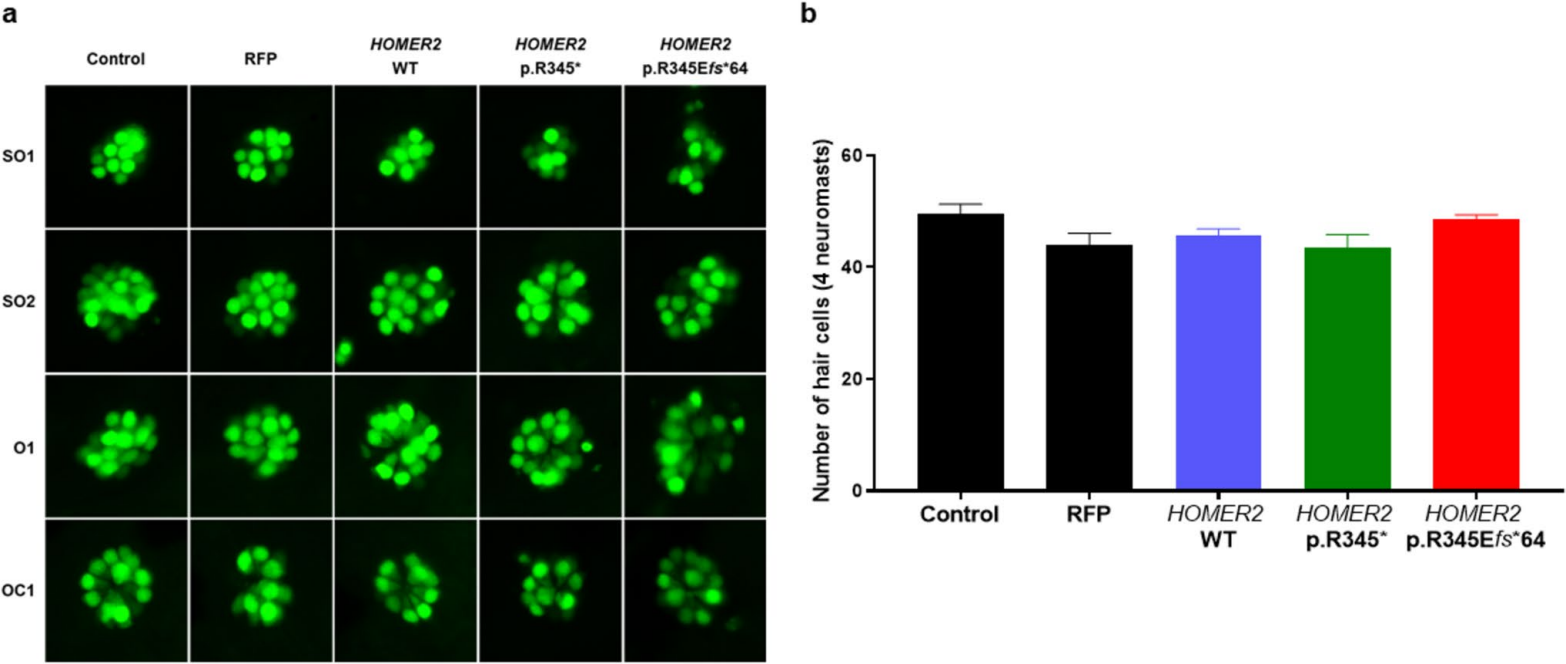
Analysis of hair cell numbers in zebrafish neuromasts. **a** Representative image of the four neuromasts (SO1, SO2, O1, and OC1) from each group: control, RFP control, *HOMER2* WT, *HOMER2* p.R345*, and *HOMER2* p.R345Efs*64. **b** Bar graph comparing each group's average number of hair cells across the four neuromasts. No statistically significant differences were observed between the groups (*p* > 0.05). *n* = 10 per group. *SO1*, supraorbital1; *SO2*, supraorbital2; *O1*, otic; *OC1*, occipital; *RFP*, red fluorescent protein; *WT*, wild-type; *R*, arginine; *E*, glutamate; *fs*, frameshift

The number of hair cells in neuromasts of zebrafish larvae was analyzed to investigate the potential impact of *HOMER2* variants on neuromast hair cell development. Hair cells from four neuromasts (supraorbital (SO1 and SO2), otic (O1), and occipital (OC1)) were examined, and the total number of hair cells was compared between groups (Fig. 5a). The statistical analysis from the four neuromasts revealed no significant differences in hair cell numbers between the groups (Fig. 5b). These results indicate that *HOMER2* variants did not affect the development of the otic capsule and the number of neuromast hair cells.

**Fig. 6:**
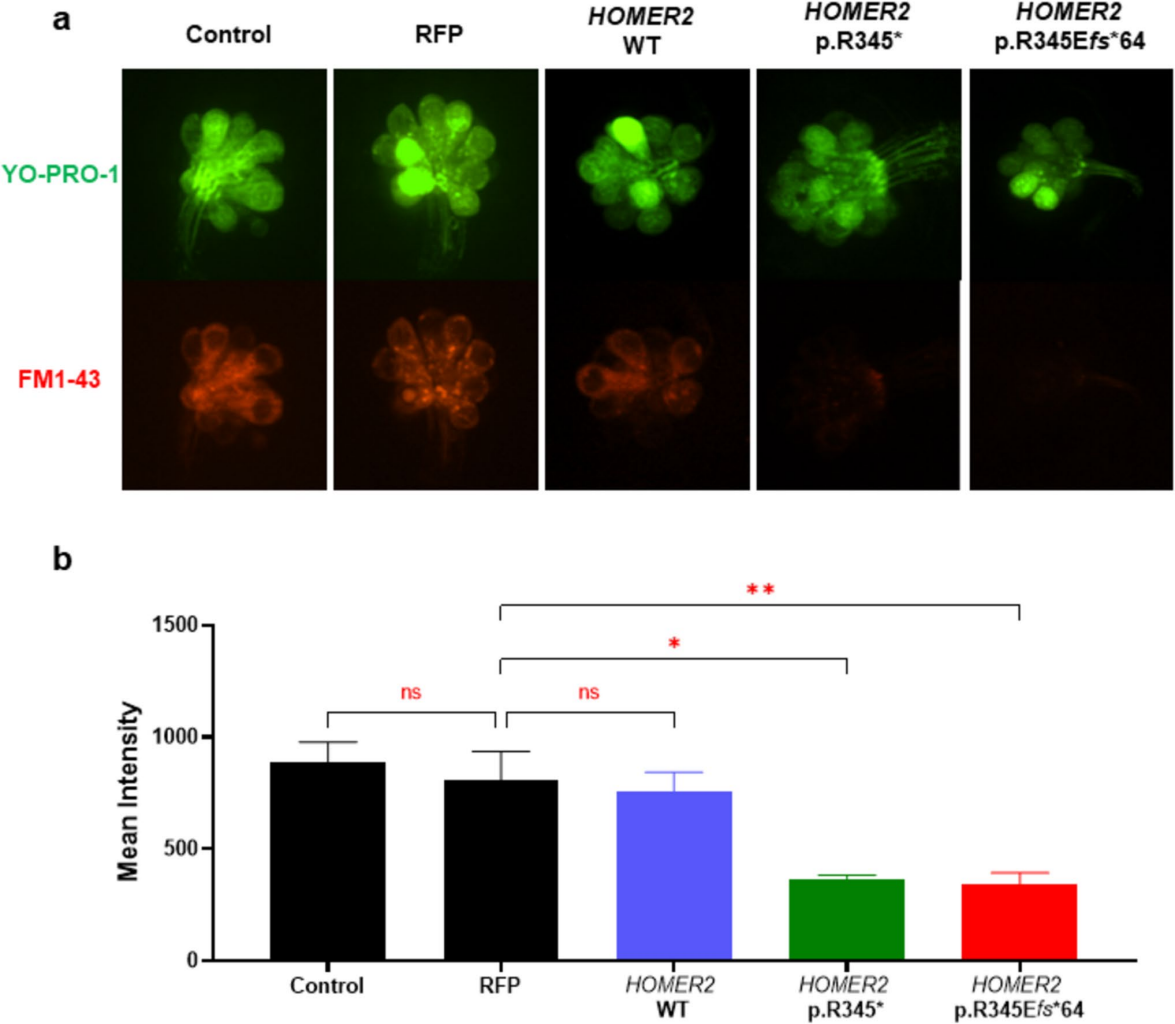
Comparison of FM1-43 uptake in zebrafish neuromast hair cells.** a** Representative image of neuromast hair cells in control, RFP control, *HOMER2* WT, and *HOMER2* variant groups. Hair cells are labeled with GFP, and FM1-43 uptake is indicated by mCherry fluorescence. **b** Bar graph comparing the fluorescence intensity of FM1-43 uptake across groups. While no significant differences were observed between the control, RFP, and *HOMER2* WT groups, the *HOMER2* variant groups showed a statistically significant reduction in intensity compared to the RFP group (*p < 0.05, **p < 0.01). *n* = 5 per group. *RFP*, red fluorescent protein; *WT*, wild-type

To evaluate the impact of *HOMER2* variants on neuromast hair cell function, FM1-43 uptake was assessed. In the control, RFP, and *HOMER2* WT groups, robust FM1-43 uptake was observed within the neuromast hair cells. However, in the *HOMER2* variant groups, FM1-43 uptake was markedly reduced (Fig. 6a). In the quantification assay, while no significant differences were detected in the mCherry fluorescence between the control, RFP, and *HOMER2* WT, the *HOMER2* variants exhibited a significant reduction in intensity compared to the RFP group (0.41-fold in p.R345* and 0.39-fold in p.R345Efs*64) (Fig. 6b). The data suggest that *HOMER2* variants severely impaired the capacity of neuromast hair cells to uptake FM1-43, indicating disrupted hair cell function in variant larvae.

**Fig. 7:**
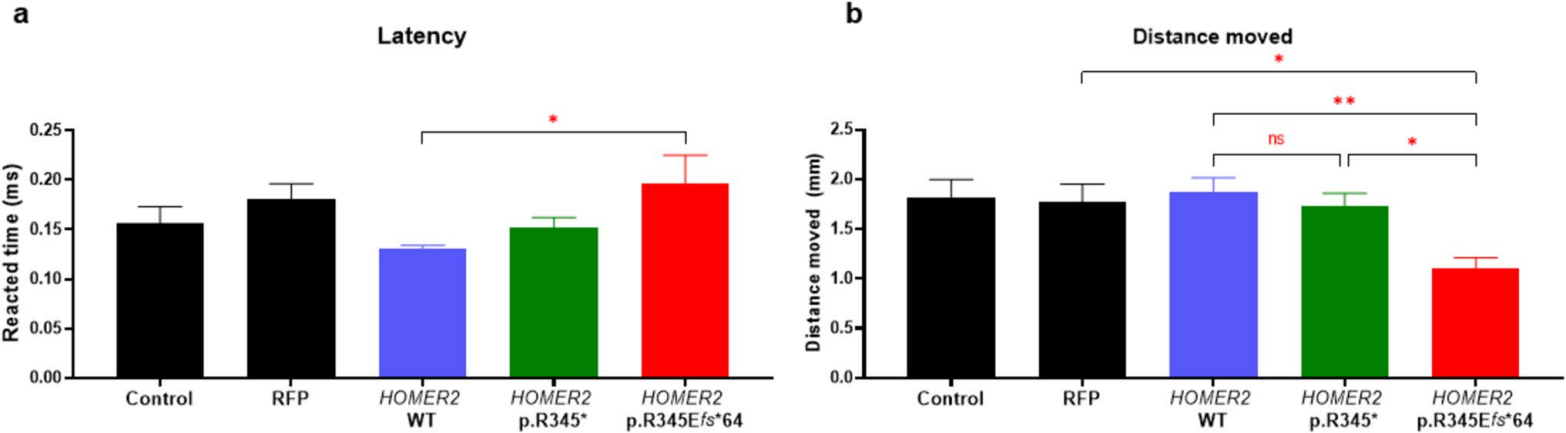
Comparison of startle reflex in zebrafish larvae at 6 days post-fertilization.** a** Bar graph depicting the latency (time from stimulus application to the onset of movement) across the control, RFP control, *HOMER2* WT, *HOMER2* p.R345*, and *HOMER2* p.R345Efs*64 groups. The latency was significantly longer in the *HOMER2* p.R345Efs*64 group compared to *HOMER2* WT (*p* < 0.05). **b** Bar graph showing the distance moved during the startle response. The *HOMER2* p.R345Efs*64 groups exhibited a significant reduction in distance moved compared to *HOMER2* WT and p.R345*, and showed a further reduction relative to the RFP control group (*p* < 0.05). *n* = 20 per group. *RFP*, red fluorescent protein; *WT*, wild-type; *R*, arginine; *E*, glutamate; *fs*, frameshift

In the light–dark behavior test, the expression of *HOMER2* variants did not alter the behavioral response of zebrafish larvae (Supplementary Fig. 2). For another behavioral test at 6 dpf, latency increased progressively in the *HOMER2* p.R345Efs*64 group (Fig. 7a). In terms of the distance moved during the startle reflex, the *HOMER2* p.R345Efs*64 group exhibited a significantly lower distance moved compared to both the *HOMER2* WT and p.R345* groups (Fig. 7b). Additionally, the distance moved in the *HOMER2* p.R345Efs*64 group was also significantly lower than that in the RFP control group, further underscoring the severe impact of the p.R345Efs*64 variant. The increased response time coupled with reduced movement suggests a functional impairment in neuromotor coordination in the *HOMER2* variant larvae. Especially, p.R345Efs*64 variant showed greater functional deficits compared to the p.R345* variant, highlighting the variant’s stronger impact on neuromotor coordination and reflex amplitude.

## Discussion

Despite its key role in the precision of calcium dynamics crucial for sound processing [1, 23], the contribution of HOMER2 to auditory function has only recently been explored, especially regarding genetic variants affecting hearing loss. This limited focus on *HOMER2* partly stems from the fact that SNHL caused by variants in this gene tends to become severe only in middle age. Genetic testing is more common for individuals with prelingual deafness or SNHL in adolescence, but less frequent for those whose hearing deteriorates in their 40 s or 50 s. Consequently, the prevalence of *HOMER2* variants may be underestimated. In patients who experience profound SNHL in their 60 s or 70 s, family history is often unclear, as their children may not yet exhibit hearing loss and their parents may have passed away, making genetic testing less likely. Thus, screening for *HOMER2* variants in patients who receive CI in their 60 s or 70 s could be beneficial [26, 27].

In one of our patients, we identified a novel frameshift extension variant (c.1033 del) in *HOMER2*, a variant type very rarely reported in the literature. Our review found that this frameshift extension variant is only the seventh *HOMER2* variant documented in patients with SNHL. Among these, two are missense variants [1, 18], one is a single nucleotide duplication [3], another is a truncation variant [2], the fifth is an in-frame deletion [17], and the sixth is a nonstop extension variant, resulting in the addition of 10 extra amino acids [4] (Table 1). Our variant, p.R345Efs*64, alters 10 amino acids at the C-terminal and adds 53 aberrant amino acids beyond the stop codon, making it the longest known translation product among *HOMER2* variants.

Previous studies have shown that truncation variants in HOMER2 can cause hearing loss through gain-of-function or dominant-negative mechanisms, even without triggering nonsense-mediated decay (NMD) [4]. RNA analysis confirmed the presence of transcripts without NMD, suggesting mechanisms independent of transcript degradation. As our variant is also in the last exon, it is likely unaffected by NMD. The combination of changes in 10 amino acids at the C-terminus and the addition of 53 aberrant amino acids likely causes a dominant-negative effect, leading to hearing loss. The recent report of a non-stop extension variant supports the idea that extension variants alone can drive hearing loss, reinforcing the hypothesis that our longer extension variant is pathogenic through a similar mechanism. To confirm this, we conducted molecular modeling and functional studies in zebrafish, comparing a hypothetical truncated variant (*HOMER2* p.R345*) with our frameshift extension variant (*HOMER2* p.R345Efs*64).

AlphaFold2 predictions suggest that the p.R345Efs*64 variant induces significant structural changes in the N-terminal EVH1 domain of HOMER2 without affecting dimer and tetramer formation. The EVH1 domain is crucial for interactions with proteins like Cdc42, and disruptions here can cause hearing loss [1, 2]. *HOMER2* variants*,* including p.R345Efs*64, may alter the Cdc42 binding structure within HOMER2-HOMER1 dimers (Supplementary Fig. 3a). In predicted models, both variants expose GDP and free magnesium (Mg), but the GDP site forms only on the p.R345Efs*64 side when dimerized with HOMER1 (Supplementary Fig. 3b). Consequently, the p.R345Efs*64 variant is expected to cause more severe structural changes, affecting both the CC and EVH1 domains and their interaction with Cdc42, leading to a greater impact on hearing than the p.R345* variant.

Zebrafish models with truncation variants exhibit structural abnormalities in the inner ear and neuromasts, while extension variants cause SNHL without obvious structural defects [1, 4]. Our variant, which combines features of truncation and extension, alters the last 10 amino acids and extends by 53 amino acids beyond the stop codon. This combination led us to investigate whether such alterations would result in the same inner ear and neuromast abnormalities seen in zebrafish models with truncation variants.

When we injected *HOMER2* p.R345* and p.R345Efs*64 mRNAs into zebrafish embryos, neither variant caused significant morphological defects in the otic capsule or neuromast hair cell counts. However, both variants significantly impaired neuromast hair cell function, as indicated by reduced FM1-43 uptake compared to controls. In the startle reflex test, both variants reduced distance and increased latency, providing strong evidence for hearing loss and the pathogenicity of the p.R345Efs*64 variant. Notably, the *HOMER2* p.R345Efs*64 variant more significantly increased the frequency of cardiac anomalies and general morphological abnormalities in zebrafish compared to both the control and the hypothetical *HOMER2* p.R345* variant. Additionally, it exacerbated the auditory phenotype, further suggesting that the extension of 53 amino acids, either alone or in conjunction with the altered C-terminal sequence, contributes to more severe hearing loss and developmental issues, including an increase in cardiac anomalies. Although this patient did not exhibit cardiac abnormalities, it is conceivable that fetuses carrying this variant in humans may experience miscarriage or early death due to multiple congenital anomalies. Nevertheless, in terms of inner ear phenotypes, such as otic capsule structure and hair cell count, our variant appears more closely aligned with extension variants than with truncation variants reported in previous literature.

In summary, we identified a HOMER2 variant classified as “likely pathogenic” based on the ACMG/AMP variant classification guidelines (Supplementary Table 3), thereby demonstrating that *HOMER2* is a causative gene in elderly patients, particularly those in their 60 s or 70 s, who develop profound SNHL and require CI. This finding highlights the need for genetic diagnosis in this older population. We also provided evidence of a pathogenic gain-of-function effect in the most C-terminal extended variant of *HOMER2* reported to date, potentially guiding future gene therapy strategies. While our frameshift extension variant aligns with known genotype–phenotype correlations, we also present novel evidence that *HOMER2* variants may be linked to systemic malformations.

## Supplementary Information

Below is the link to the electronic supplementary material.

ESM 1 (DOCX 786 KB)

ESM 2 (DOCX 39.9 KB)

## Data Availability

The datasets generated during the current study are available in this published article.
