# Supplementary material for: Impact of HOMER2 frameshift extension variant on auditory function and development
Source: J Mol Med (Berl). 2025 Jun 14;103(8):975–87. doi: 10.1007/s00109-025-02556-7 (PMC12343744; doi:10.1007/s00109-025-02556-7)
Supplement: Supplementary file 1 — (DOCX 786 KB) [file 109_2025_2556_MOESM1_ESM.docx]

**Supplementary Materials**

**Supplementary Fig. 1**. Variant filtering strategy of exome sequencing data

**Initial variant calls**

(SNVs, indels)

**Nonsynonymous or splice site variants**

**with read depth >= 20**

(Including exonic and +/-8 intronic variants)

**Rare variants <= 0.002**

(1000G, ESP6500)

**Sorting data based on known deafness genes**

(about 400 genes)

**dbSNP/ClinVar database: allele frequence**

(GMAF <= 0.002)

**- Inheritance pattern, phenotype matching**

(OMIM, HHL)

**- In silico prediction, conservation**

(by the CADDphred, and REVEL)

**- Omitting gene**

(not involved in the pathogenesis on clinical phenotype)

**Genetic correlation analysis**

(Clinical features, Audiogram profile)

**Total variants**

**124,743**

**37,862**

**Number of candidate variants in SB1190-1923**

**2,839**

**35**

**8**

**1**

**(*HOMER2*)**

**1**

**(*HOMER2*)**

Genetic diagnosis flow. Multiple bioinformatics tools were adopted to narrow down the candidate causative variants, and then clinical information including inheritance pattern/online genotype-phenotype databases, was used to finalize the genetic diagnosis. SNV, single nucleotide variant; Indel, insertion-deletion; 1000G, 1000 Genomes Project; ESP6500, Exome Sequencing Project v. 6500; GMAF, Global minor allele frequency; OMIM, Online Mendelian Inheritance in Man; HHL, Hereditary Hearing Loss homepage; CADD, Combined Annotation Dependent Depletion; REVEL, Rare Exome Variant Ensemble Learner

*Web Resources.*

CADD: <https://cadd.gs.washington.edu/>, REVEL: <https://sites.google.com/site/revelgenomics/>, KOVA: <https://www.kobic.re.kr/kova/>, GnomAD: <https://gnomad.broadinstitute.org/>, HHL: <https://hereditaryhearingloss.org/>, OMIM: <https://www.omim.org/>

**The light-dark behavior test**

The light-dark behavior of zebrafish larvae was evaluated at 6 days post-fertilization after mRNA injection of *HOMER2* WT and its variants (p.R345* and p.R345E*fs**64), with uninjected and RFP control groups used for comparison (**Supplementary Fig. 2a**). Larvae in all groups exhibited similar movement patterns during the light-off phase, characterized by a typical increase in locomotor activity when the lights were turned off. Similarly, during the light-on phase, all groups showed a decrease in activity, which is expected in response to light exposure. The *HOMER2* WT and variant groups did not display any aberrant locomotor behavior compared to the controls during either phase.

Quantification of the total distance moved during the 5-minute light-off and light-on phases revealed no statistically significant differences among the groups (p > 0.05) (**Supplementary Fig. 2b**). These results indicate that the expression of *HOMER2* variants did not alter the behavioral response of zebrafish larvae to changes in light conditions at this stage of development, with all groups displaying normal locomotor patterns in response to light-dark cycles.


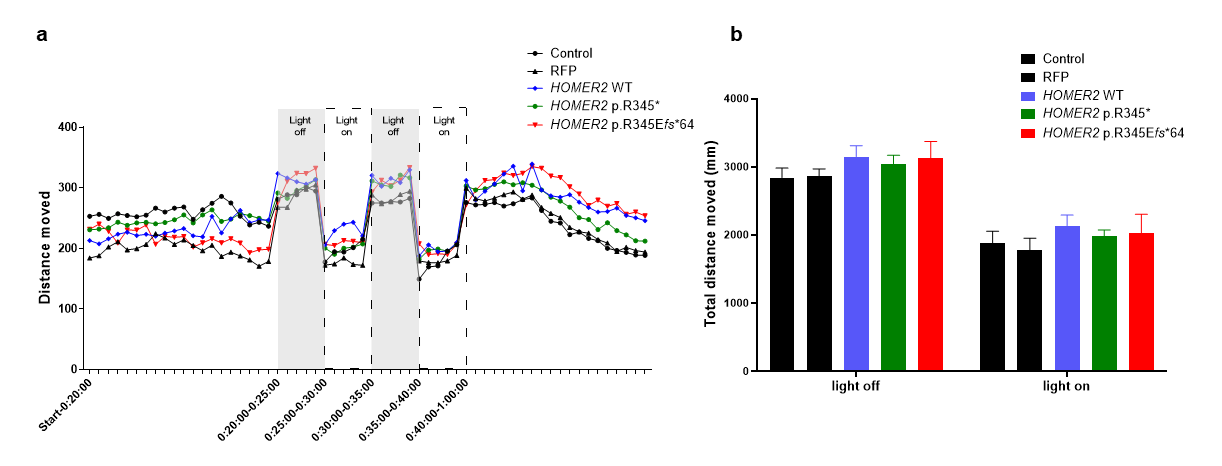


**Supplementary Fig. 2. Light-dark behavior test in zebrafish larvae at 6 days post-fertilization.** **a** Graph showing the movement trajectories of zebrafish larvae during the light-dark behavior test. The test included two 5-minute cycles of light-off and light-on phases following a 20-minute adaptation period. **b** Bar graph comparing the total distance moved during the 5-minute light-off and 5-minute light-on periods across the control, RFP control, *HOMER2* WT, *HOMER2* p.R345*, and *HOMER2* p.R345E*fs**64 groups. No statistically significant differences were observed between the groups in either the light-off or light-on phases (p > 0.05). n=20 per each group


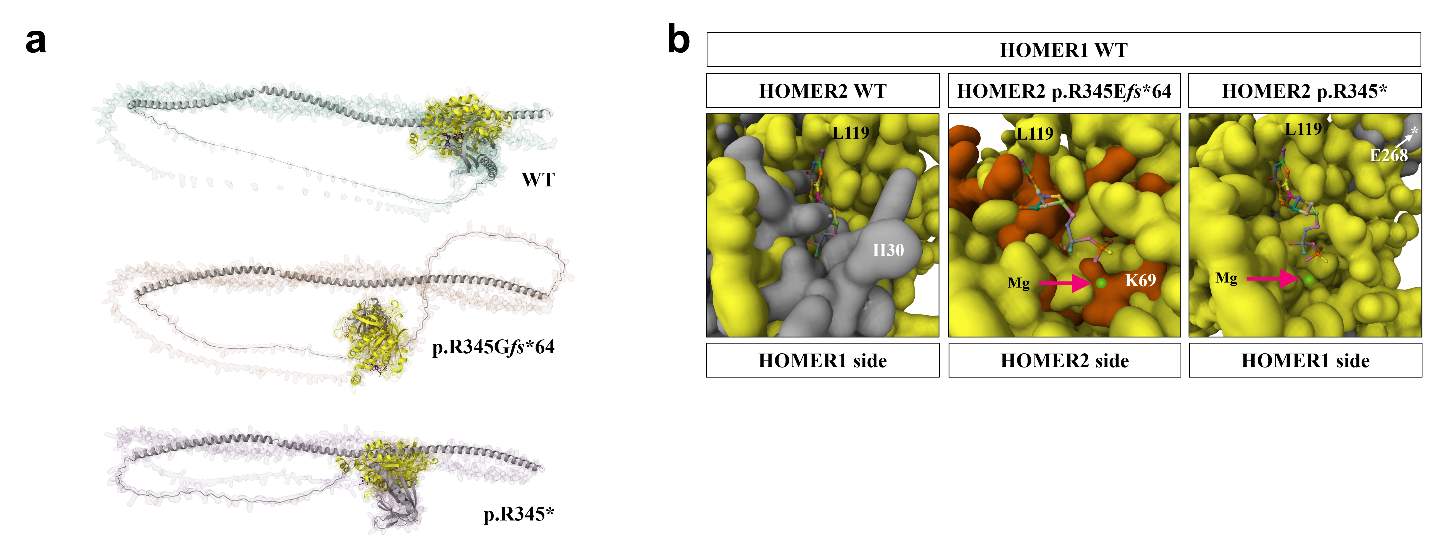


**Supplementary Fig. 3. Predicted 3D structures of HOMER dimer and Cdc42 interaction were obtained using AlphaFold2.** **a** The prediction of interacted Cdc42 (yellow) with HOMER1 and HOMER2 WT or HOMER2 variants is also shown. HOMER1 is presented with a gray colored structure, and HOMER2 WT, HOMER2 p.R345E*fs**64, and HOME2 p.R345* are presented with green, brown, and purple colors, respectively. **b** The predicted localization of GDP (colored using Atom Id in Mol*3D Viewer) and Mg (yellow green ball) at the pocket of Cdc42-dimer and HOMER1 WT-each of HOMER2 WT or variants dimer structure is illustrated.
