# Supplementary material for: Impact of HOMER2 frameshift extension variant on auditory function and development
Source: J Mol Med (Berl). 2025 Jun 14;103(8):975–87. doi: 10.1007/s00109-025-02556-7 (PMC12343744; doi:10.1007/s00109-025-02556-7)
Supplement: Supplementary file 2 — (DOCX 39.9 KB) [file 109_2025_2556_MOESM2_ESM.docx]

**Supplementary table 1.** List of 34 genes filtered out from whole exome sequencing (Benign/VUS)

| **Locus (OMIM)** | **Gene (OMIM)** | **Genomic Position: Change (GRCh37/hg19)** | **Transcript Isoform** | **Nucleotide**  **change** | **Amino Acid**  **change** | **Zygosity** | **Effect** | **Gene-Phenotype Relationships**  **(OMIM)** | | **Alternative Allele Frequency** | | **ACMG/AMP 2018 guideline** | | **ClinVar/ dbSNP155** |
| --- | --- | --- | --- | --- | --- | --- | --- | --- | --- | --- | --- | --- | --- | --- |
|  |  |  |  |  |  |  |  | **Phenotype** | **Disease Inheritance** | **KOVA** | **GMAF**  **(gnomAD)** | **Criteria** | **Classification** |  |
| **DFNB44** | **ADCY1** | **Chr7:45756839 C>T** | **NM_021116.2** | **c.*3245C>T** |  | **Heterozygote** | **3 prime UTR variant** | **prelingual, profound hearing loss** | **AR** | **T=0.0010 (3/2922, KOREAN)** | **T=0.000080 (12/149204, GnomAD_genomes)** | **BA1** | **Benign** | **Not Reported/**  **rs551333410** |
| **DFNA7/49** | **ATP1A2** | **Chr1:160111230 A>G** | **NM_000702.3** | **c.*118A>G** |  | **Heterozygote** | **3 prime UTR variant** | **progressive hearing loss, familial hemiplegic migraine-2** | **AD** | **G=0.0005 (1/1832, Korea1K)** | **G=0.0000018 (2/1131790, GnomAD_exomes)** | **PM2** | **VUS** | **Not Reported/**  **rs1570999487** |
| DFNB47 | ATP6V1C2 | Chr2:10924686 CT>C | NM_001039362.1 | c.*1299delT |  | Heterozygote | 3 prime UTR variant | prelingual hearing loss | AR | delT=0.4093 (2961/7234, Korea4K) | TT=0.485308 (153968/317258, GnomAD_exomes) | BA1 | Benign | Not Reported/ rs35148273 |
| DFNB6 | CACNA1D | Chr3:53779647 A>G | NM_000720.3 | c.3067-4G>A |  | Heterozygote | splice region variant | sinoatrial node dysfunction and congenital deafness | AR | A=0.0008 (6/7234, Korea4K) | A=0.0000920 (128/1391608, GnomAD_exomes) | BS1_Supporting | Benign | Likely benign/ rs371031025 |
| 11q23.3 | CBL | Chr11:119171050 T>TC | NM_005188.3 | c.*559_*560insC |  | Heterozygote | 3 prime UTR variant | Noonan syndrome and conductive hearing loss | AR | insC=0.4034 (2918/7234, Korea4K) | insC=0.267311 (64670/241928, GnomAD_exomes) | BA1 | Benign | Likely benign/ rs3833768 |
| 11q23.3 | CBL | Chr11:119171051 A>AAT | NM_005188.3 | c.*560_*561insAT |  | Heterozygote | 3 prime UTR variant | Noonan syndrome and conductive hearing loss | AR | insAT=0.4034 (2918/7234, Korea4K) | insAT=0.243316 (36201/148782, GnomAD_genomes) | BA1 | Benign | Likely benign/ rs56722042 |
| **DFNB32/105** | **CDC14A** | **Chr1:100964651 G>A** | **NM_033312.2** | **c.1588G>A** | **p.Glu530Lys** | **Heterozygote** | **missense variant** | **moderate to profound, prelingual onset of deafness** | **AR** | **A=0.0005 (1/1832, Korea1K)** | **Absent** | **PM2** | **VUS** | **Not Reported/**  **rs1571348744** |
| 15q26.3 | CHSY1 | Chr15:101716007 C>CT | NM_014918.4 | c.*1585_*1586insA |  | Homozygote | 3 prime UTR variant | Temtamy preaxial brachydactyly syndrome | AR | -=0.1042 (754/7234, Korea4K) | -=0.322892 (48127/149050, GnomAD_genomes) | BA1 | Benign | Not Reported/ rs35004007 |
| 4q24 | CISD2 | Chr4:103809211 CTGTG>CTG | NM_001008388.4 | c.*652_*653delGT |  | Heterozygote | 3 prime UTR variant | Wolfram syndrome | AR | no data | delGT=0.010 (2/202, GnomAD_exomes) | BA1 | Benign | Not Reported/ rs35045951 |
| 4q24 | CISD2 | Chr4:103810431 A>T | NM_001008388.4 | c.*1844A>T |  | Heterozygote | 3 prime UTR variant | Wolfram syndrome | AR | T=0.4589 (1341/2922, KOREAN) | no data | BA1 | Benign | Not Reported/ rs62327288 |
| **STL5** | **COL9A2** | **Chr1:40771433 G>A** | **NM_001852.3** | **c.1061C>T** | **p.Pro354Leu** | **Heterozygote** | **missense variant** | **Stickler syndrome type V** | **AR** | **A=0.0007 (5/7234, Korea4K)** | **A=0.000074 (11/149298, GnomAD_genomes)** | **BS1_Supporting** | **Benign** | **Uncertain significance/ rs201772619** |
| Xp21.2 | DMD | ChrX:31138983 T>TATCA | NM_004006.2 | c.*1049_*1052dupTGAT |  | Heterozygote | 3 prime UTR variant | profound sensorineural hearing loss | XLR | no data | dupTCAA=0.05320 (779/14644, ALFA) | BA1 | Benign | Uncertain significance/ rs71860126 |
| DFNA39 | DSPP | Chr4:88535832 A> ATAGCAGTGACAGCAGCAG | NM_014208.3 | c.2036_2053dupGTAGCAGTGACAGCAGCA | p.Ser679_Ser684dup | Homozygote | disruptive inframe insertion | dentinogenesis imperfecta type 1 and deafness | AD | dupGTAGCAGTGA(CAG)2CA=0.4880 (3529/7232, Korea4K) | dupGTAGCAGTGA(CAG)2CA=0.28477 (5757/20216, ExAC) | BA1 | Benign | Benign/ rs1553904159 |
| 20q13 | EDN3 | Chr20:57899653 AC>A | NM_207032.1 | c.*241delC |  | Heterozygote | 3 prime UTR variant | Waardenburg syndrome, type 4B | AD or AR | no data | delC=0.05945 (765/12868, ALFA) | BA1 | Benign | Benign/ rs11475273 |
| 18q12.3-q21.1 | EPG5 | Chr18:43429751 C>T | NM_020964.2 | c.*2681G>A |  | Heterozygote | 3 prime UTR variant | Vici syndrome | AR | T=0.0036 (26/7232, Korea4K) | T=0.000100 (15/149286, GnomAD_genomes) | BS1 | Benign | Not Reported/ rs76336249 |
| **18q12.3-q21.1** | **EPG5** | **Chr18:43431320 A>G** | **NM_020964.2** | **c.*1112T>C** |  | **Heterozygote** | **3 prime UTR variant** | **Vici syndrome** | **AR** | **G=0.0010 (3/2922, KOREAN)** | **G=0.000007 (1/149230, GnomAD_genomes)** | **BS1_Supporting** | **Benign** | **Not Reported/ rs1261270913** |
| DFNA10 | EYA4 | Chr6:133850246 AC>A | NM_001301013.1 | c.*306delC |  | Homozygote | 3 prime UTR variant | Deafness, sensorineural, progressive | AD | no data | delC=0.370263 (55130/148894, GnomAD_genomes) | BA1 | Benign | Not Reported/ rs3842093 |
| 4p16.3 | FGFR3 | Chr4:1809127 CGT>C | NM_001163213.1 | c.*156_*157delTG |  | Homozygote | 3 prime UTR variant | LADD syndrome 2 | AD | no data | delTGTG=0.14703 (2295/15609, ALFA) | BA1 | Benign | Benign/ rs34562534 |
| 10p14 | GATA3 | Chr10:8116241 G>GA | NM_001002295.1 | c.*265dupA |  | Homozygote | 3 prime UTR variant | Hypoparathyroidism, sensorineural deafness, and renal dysplasia | AD | no data | (A)10=0.2526 (1265/5008, 1000G) | BA1 | Benign | Benign/ rs3839918 |
| DFNA28 | GRHL2 | Chr8:102643941 A>G | NM_024915.3 | c.1334A>G | p.Gln445Arg | Heterozygote | missense variant | Deafness, sensorineural | AD | G=0.0010 (7/7234, Korea4K) | G=0.0000336 (47/1400432, GnomAD_exomes) | BS1_Supporting | Benign | Not Reported/ rs145717789 |
| DFNA28 | GRHL2 | Chr8:102679284 A>G | NM_024915.3 | c.*353A>G |  | Heterozygote | 3 prime UTR variant | Deafness, sensorineural | AD | G=0.0010 (7/7234, Korea4K) | G=0.000068 (13/191040, GnomAD_exomes) | BS1_Supporting | Benign | Not Reported/ rs137912492 |
| DFNB39 | HGF | Chr7:81380812 T>TG | NM_001010934.1 | c.*615_*616insC |  | Heterozygote | 3 prime UTR variant | Deafness, sensorineural, prelingual, profound,  Nonprogressive deafness | AR | -=0.1535 (1110/7232, Korea4K) | -=0.176022 (26203/148862, GnomAD_genomes) | BA1 | Benign | Not Reported/ rs11404675 |
| DFNB39 | HGF | Chr7:81380814 CATAGACA>C | NM_001010934.1 | c.*607_*613delTGTCTAT |  | Heterozygote | 3 prime UTR variant | Deafness, sensorineural, prelingual, profound,  Nonprogressive deafness | AR | ATAGACA=0.1533 (1108/7228, Korea4K) | ATAGACA=0.235775 (189545/803924, GnomAD_exomes) | BA1 | Benign | Not Reported/ rs373999040 |
| 1p36.11 | IFNLR1 | Chr1:24481706 ATG>A | NM_170743.3 | c.*1912_*1913delCA |  | Homozygote | 3 prime UTR variant | nonsyndromic, progressive sensorineural hearing loss | AD | (TG)7=0.0039 (28/7230, Korea4K) | (TG)7=0.21 (16/76, GnomAD_exomes) | BS1 | Benign | Not Reported/ rs35747193 |
| 20p12.2 | JAG1 | Chr20:10621771 T>A | NM_000214.2 | c.3038A>T | p.His1013Leu | Heterozygote | missense variant | Deafness (mild-to-severe, primarily affecting middle frequencies), Vestibular dysfunction | AD | A=0.0076 (55/7234, Korea4K) | A=0.000047 (7/149206, GnomAD_genomes) | BA1 | Benign | Likely benign/ rs758687380 |
| **Xp22.31** | **KAL1** | **ChrX:8498309 G>A** | **NM_000216.2** | **c.*2727C>T** |  | **Heterozygote** | **3 prime UTR variant** | **Hypogonadotropic hypogonadism 1 with or without anosmia (Kallmann syndrome 1)** | **XLR** | **A=0.0031 (16/5215, Korea4K)** | **A=0.000055 (6/109444, GnomAD_genomes)** | **BS1** | **Benign** | **Not Reported/ rs1270685169** |
| 3q27.3 | MASP1 | Chr3:186952034 GA>G | NM_139125.3 | c.*1437delT |  | Homozygote | 3 prime UTR variant | 3MC syndrome 1, conductive hearing loss | AR | A=0.0018 (13/7234, Korea4K) | A=0.0820434 (89711/1093458, GnomAD_exomes) | BA1 | Benign | Not Reported/ rs770168258 |
| 3q27.3 | MASP1 | Chr3:186952037 A>AG | NM_139125.3 | c.*1434_*1435insC |  | Homozygote | 3 prime UTR variant | 3MC syndrome 1, conductive hearing loss | AR | -=0.0018 (13/7234, Korea4K) | -=0.0820454 (89715/1093480, GnomAD_exomes) | BA1 | Benign | Not Reported/ rs763002473 |
| **12p12.3** | **MGP** | **Chr12:15035134 A>G** | **NM_001190839.1** | **c.326T>C** | **p.Met109Thr** | **Heterozygote** | **missense variant** | **Keutel syndrome, Hearing loss (sensorineural, mixed, and conductive)** | **AR** | **G=0.0010 (3/2922, KOREAN)** | **G=0.000016 (2/121398, ExAC)** | **BS1_Supporting** | **Benign** | **Not Reported/ rs752029154** |
| 22q11.21 | PEX26 | Chr22:18571643 A>G | NM_001127649.2 | c.*802A>G |  | Heterozygote | 3 prime UTR variant | sensorineural hearing loss and Amelogenesis imperfecta | AR | G=0.0019 (14/7234, Korea4K) | G=0.000011 (3/264690, TOPMED) | BS1_Supporting | Benign | Uncertain significance/ rs1046921426 |
| 17p11.2 | RAI1 | Chr17:17697098 AGC>A | NM_030665.3 | c.837_838delGC | p.Gln279fs | Homozygote | frameshift variant | Smith-Magenis syndrome, Hearing loss (conductive and/or sensorineural) | AD | GC=0.1904 (1375/7220, Korea4K) | delGC=0.3853704 (484873/1258200, GnomAD_exomes) | BA1 | Benign | Benign/ rs35068024 |
| 17p11.2 | RAI1 | Chr17:17697101 AG>A | NM_030665.3 | c.840delG | p.Gln280fs | Homozygote | frameshift variant | Smith-Magenis syndrome, Hearing loss (conductive and/or sensorineural) | AD | no data | delG=0.3245652 (426036/1312636, GnomAD_exomes) | BA1 | Benign | Benign/ rs34083643 |
| DFNB115 | SPNS2 | Chr17:4439727 T>TG | NM_001124758.1 | c.1607+6_1607+7insG |  | Heterozygote | splice region variant | Hearing loss, sensorineural, severe, Absent acoustic reflexes | AR | dupG=0.1276 (212/1662, Korea1K) | dupG=0.43500 (4745/10908, GO-ESP) | BA1 | Benign | Benign/ rs3053652 |
| DFNB6 | TMIE | Chr3:46751073 TAAG>T | NM_147196.2 | c.391_393delAAG | p.Lys131del | Homozygote | inframe deletion | Congenital or prelingual onset hearing loss, severe to profound | AR | no data | del(AAG)3=0.0061 (7/1142, 1000G) | BA1 | Benign | Benign/ rs10578999 |

- dbSNP/ClinVar database : allele frequence (GMAF <= 0.002) : 7 (bold)

**Supplementary table 2. Amino acid sequences of HOMER2 WT and its variants**

| HOMER2 | length |
| --- | --- |
| **WT**  MGEQPIFTTRAHVFQIDPNTKKNWMPASKQAVTVSYFYDVTRNSYRIISVDGAKVIINSTITPNMTFTKTSQKFGQWADSRANTVFGLGFSSEQQLTKFAEKFQEVKEAAKIAKDKTQEKIETSSNHSQESGRETPSSTQASSVNGTDDEKASHAGPANTHLKSENDKLKIALTQSAANVKKWEIELQTLRESNARLTTALQESAASVEQWKRQFSICRDENDRLRNKIDELEEQCSEINREKEKNTQLKRRIEELEAELREKETELKDLRKQSEIIPQLMSECEYVSEKLEAAERDNQNLEDKVRSLKTDIEESKYRQRHLKVELKSFLEVLDGKIDDLHDFRRGLSKLGTDN | 354 |
| **p.R345E*fs**64**  MGEQPIFTTRAHVFQIDPNTKKNWMPASKQAVTVSYFYDVTRNSYRIISVDGAKVIINSTITPNMTFTKTSQKFGQWADSRANTVFGLGFSSEQQLTKFAEKFQEVKEAAKIAKDKTQEKIETSSNHSQESGRETPSSTQASSVNGTDDEKASHAGPANTHLKSENDKLKIALTQSAANVKKWEIELQTLRESNARLTTALQESAASVEQWKRQFSICRDENDRLRNKIDELEEQCSEINREKEKNTQLKRRIEELEAELREKETELKDLRKQSEIIPQLMSECEYVSEKLEAAERDNQNLEDKVRSLKTDIEESKYRQRHLKVELKSFLEVLDGKIDDLHDFREGSPSWAPITRAGRGPGPARESQACVRDQIALGRSSVCIASVNAGAVCRVSKPVVPSTHSFSE | 407 |
| **p.R345***  MGEQPIFTTRAHVFQIDPNTKKNWMPASKQAVTVSYFYDVTRNSYRIISVDGAKVIINSTITPNMTFTKTSQKFGQWADSRANTVFGLGFSSEQQLTKFAEKFQEVKEAAKIAKDKTQEKIETSSNHSQESGRETPSSTQASSVNGTDDEKASHAGPANTHLKSENDKLKIALTQSAANVKKWEIELQTLRESNARLTTALQESAASVEQWKRQFSICRDENDRLRNKIDELEEQCSEINREKEKNTQLKRRIEELEAELREKETELKDLRKQSEIIPQLMSECEYVSEKLEAAERDNQNLEDKVRSLKTDIEESKYRQRHLKVELKSFLEVLDGKIDDLHDFR | 344 |

**Supplementary table 3. ACMG/AMP variant classification summary report for HOMER2 variant**

| **Variant Information** | |
| --- | --- |
| Gene:  Transcript:  Genomic Location:  Nucleotide Change:  Protein Change:  Variant Type:  Inheritance Pattern: | HOMER2  NM_199330.2  Chr15:83518498 (GRCh37/hg19)  c.1033del  p.Arg345GlufsTer64  Frameshift + stop-loss → C-terminal elongation  Autosomal dominant (related to deafness) |
| **Population Data** | |
| This variant is not observed in large population cohorts (gnomAD, ExAC, 1000 Genomes).  → **PM2: Moderate evidence strength** | |
| **Functional Data** | |
| Functional studies using zebrafish models demonstrate that the mutant form exhibits a pathogenic phenotype compared to wild-type.  → **PS3: Moderate evidence strength** | |
| **Protein Impact** | |
| The variant leads to a frameshift at codon 345, removing the natural stop codon and producing a novel protein sequence with an additional 64 amino acids at the C-terminus.  → **PM4: Moderate evidence strength** | |
| **Criteria Applied** | |
| \| **Criterion** \| **Strength** \| **Rationale** \| \| --- \| --- \| --- \| \| PS3 \| Moderate \| Zebrafish functional assay shows pathogenic phenotype \| \| PM2 \| Moderate \| Absent from population databases \| \| PM4 \| Moderate \| Stop-loss with extended abnormal protein length \| | |
| **Final Classification** | |
| ***Likely Pathogenic*** | |
